# A new fluoroscopic view for evaluation of anteromedial cortex reduction quality during cephalomedullary nailing for intertrochanteric femur fractures: the 30° oblique tangential projection

**DOI:** 10.1186/s12891-020-03668-6

**Published:** 2020-11-05

**Authors:** Shi-Yi Chen, Shi-Min Chang, Rujan Tuladhar, Zhen Wei, Wen-Feng Xiong, Sun-Jun Hu, Shou-Chao Du

**Affiliations:** Department of Orthopaedic Surgery, Yangpu Hospital, Tongji University School of Medicine, Shanghai, China

**Keywords:** Pertrochanteric fracture, Anteromedial cortex, Cortical apposition, Fluoroscopy, Oblique view, 3D-CT

## Abstract

**Background:**

Anteromedial cortex-to-cortex reduction is a key parameter for stable reconstruction of the fracture fragments during the intertrochanteric fracture fixation. This paper introduces the oblique fluoroscopic projection as a novel method to evaluate the quality of anteromedial cortical apposition.

**Methods:**

Three proximal femur specimens were marked with steel wires along five anatomic landmarks: Greater trochanter, Lesser trochanter, Intertrochanteric line, Anterolateral tubercle and the Anteromedial cortical line. After obtaining the standard femoral neck AP and lateral fluoroscopic images, the C-arm was rotated by every 5°increments until a clear tangential view of the antero-medial-inferior corner cortex was observed. 98 cases of intertrochanteric hip fractures were enrolled from April 2018 to October 2019. After fixation with the nails, the intra-operative anteromedial cortex reduction quality was evaluated from the AP, the true lateral, and the new anteromedial oblique fluoroscopic images. The fluoroscopic results were compared with the post-operative 3D-CT reconstruction images.

**Results:**

The specimen study showed that internal rotation of the C-arm to approximately 30 ° can remove all the obscure shadows and clearly display the antero-medial-inferior cortical tangent line. Clinically,the positive, neutral and negative apposition of different cortices via intra-operative fluoroscopic images showed79, 19 and 0 cases of medial cortical apposition in AP views; 2, 68 and 28 cases of anterior cortices in lateral views;and 22, 51 and 25cases of anteromedial cortical apposition in oblique views respectively. The post-operative 3D-CT reconstruction images revealed that the final anteromedial cortical contact was noted in 62 cases (63.3%), and lost in 36 cases (36.7%). The overall coincidence rate between intra-operative fluoroscopy and post-operative 3D-CT was 63.3% (62/98) in AP view,79.6% (78/98) in lateral view, and 86.7% (85/98) in oblique view(*p* < 0.001). Negative cortical apposition in oblique view was highly predictive of a final loss of cortical support on 3D CT (24/25 cases, 96%).And non-negative cortical apposition in oblique view was highly associated with true cortical support on 3D CT images (61/73 cases, 83.6%) (*p* < 0.001).

**Conclusions:**

Besides the AP and lateral projections, an anteromedial oblique view of 30° certifies to be a very useful means for evaluation of the fracture reduction quality of anteromedial cortical apposition.

## Background

Geriatric hip fracture cases are presumed to reach around 4.5 million worldwide by the year 2050. The per/inter-trochanteric femur fracture is the most life-threatening fracture in the elderly which is also called the last fracture of the life, with mortality rate of about 10% in 30 days, 20% in three months and 30%in one year [1]. Surgical treatment with intramedullary nail is recommended, especially in the unstable fracture patterns [2]; A variety of intramedullary nails are available in clinic [3], but the postoperative complications are still noted high [4].

The quality of the fracture reduction is the first pre-requisite in the treatment of pertrochanteric fracture. In 2015, Chang et al. [5] firstly introduced the concept of anteromedial cortical support reduction, which allowed the head-neck fragment contact with the anteromedial cortex of the femoral shaft via limited and controlled sliding. As the reduction of the displaced lesser trochanter is difficult in the unstable 31A2 pertrochanteric fracture type, the cortex-to-cortex apposition which stands as a non-anatomic functional buttress reduction is an important element to effectively support the head-neck fragment, i.e. the anteromedial cortices of the inferior corner become the key position for the cortex-to-cortex support and fracture reduction.

Currently in clinical practice, the standard femoral neck antero-posterior (AP) and lateral views are used as intraoperative fluoroscopic evaluation means to observe and judge the reduction quality of the fracture. But the clear knowledge on the view of the tangent position and adequate reduction of the anteromedial cortices of the inferior corner is still lacking. The aim of this study was to introduce a new method of anteromedial oblique fluoroscopic view, which can be used to judge the quality of anteromedial cortical reduction in pertrochanteric fractures, and to signify its value in clinical applications.

## Methods

### Three types of anteromedial cortex apposition

According to the criteria proposed by Chang et al., there are three reduction types of the anteromedial cortex: (1) Positive apposition: the anteromedial cortex of the head-neck fragment located slightly superomedial to the anteromedial cortex of the femoral shaft (less than one cortical thickness) which is also called as an extra-medullary cortex position, (2) Neutral apposition: a smooth cortical apposition of the anteromedial aspect of the two cortices, also can be called as fluoroscopic anatomic position and (3) Negative apposition: the anteromedial cortex of the head-neck fragment located superolateral to the anteromedial cortex of the shaft, which is also called as an intra-medullary cortex position.

**Fig. 1:**
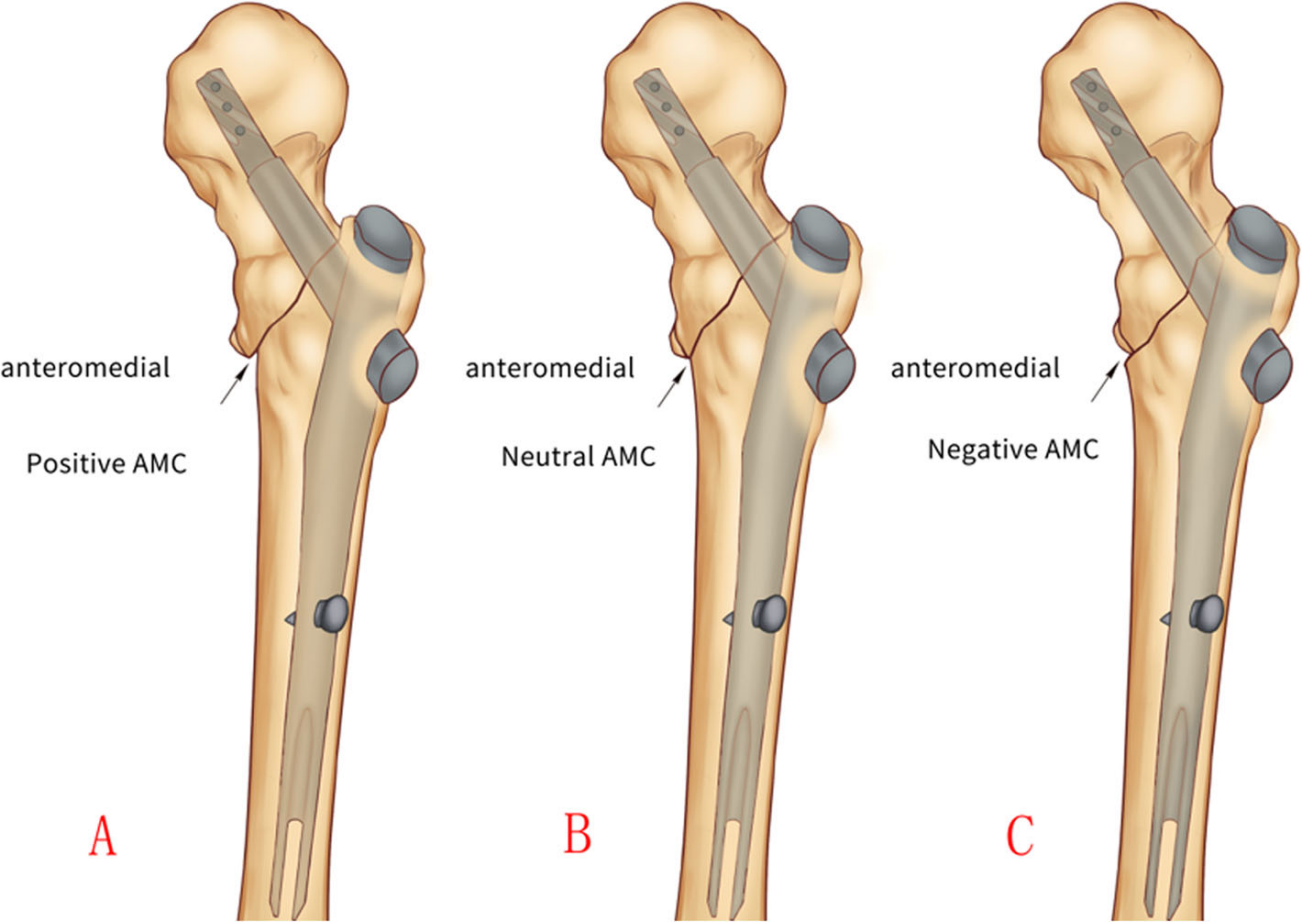
Schematic drawing to show three types of cortical appositions of the anteromedial cortices in oblique tangential view. **a**. Positive; **b**. Neutral; **c**. Negative

In the oblique fluoroscopic view, anteromedial cortex apposition was also classified into three categories, i.e. positive, neutral, and negative (Fig. 1).

### Experimental fluoroscopic study using specimens

Three proximal femur specimens were marked with steel wires over the five anatomic landmarks: the Greater trochanter, the Lesser trochanter, the Intertrochanteric line, the Anterolateral tubercle, and the Anteromedial cortical tangent line. Two observers who have been already familiar with the anatomical markings observed under fluoroscopy were present for the evaluation during the study. After obtaining the standard femoral neck AP and lateral views under the C-arm fluoroscopy, the machine was adjusted at every 5° increment until the complete elimination of the overlapping shadow of the anterior aspect was attained to finally obtain a clear tangential view of the anteromedial cortex.

### Clinical patients

After Institutional Review Board approval (LL-2017-ZRKX-013), 98 patients with sustained intertrochanteric hip fractures from April 2018 to October 2019 were prospectively included in the study. There were 42 males and 56 females, with an average age of 79.6 years (range 61–97 years). AO Foundation and Orthopaedic Trauma Association (AO/OTA) classification revealed 10 cases of type A1, 74 cases of type A2, and 14 cases of type A3.

Inclusion criteria were as follows: (1) age at or over 60 years, (2) fresh trochanteric femur fractures, (3) closed reduction and intramedullary nail fixation and (4) complete intraoperative fluoroscopy images and postoperative 3D-CT scanning. Exclusion criteria were: (1) age younger than 60 years, (2) subtrochanteric fractures and (3) pathological fractures.

After eliminating the surgical contraindication, closed reduction and nail fixation was performed on fracture traction table by senior attending orthopedic trauma surgeons. If the fracture reduction quality by closed maneuver was not of satisfactory value, then the minimal open reduction with instruments such as bone hook was performed. All fractures were fixed with proximal femoral nail anti-rotation system.

The C-arm machine was routinely placed in the crotch between the two legs, and was used to control the operation, including the fracture reduction quality (Garden alignment and cortex apposition in AP, lateral and oblique views) and the implant positioning (TAD and Parker ratio in AP and lateral views).

**Fig. 2:**
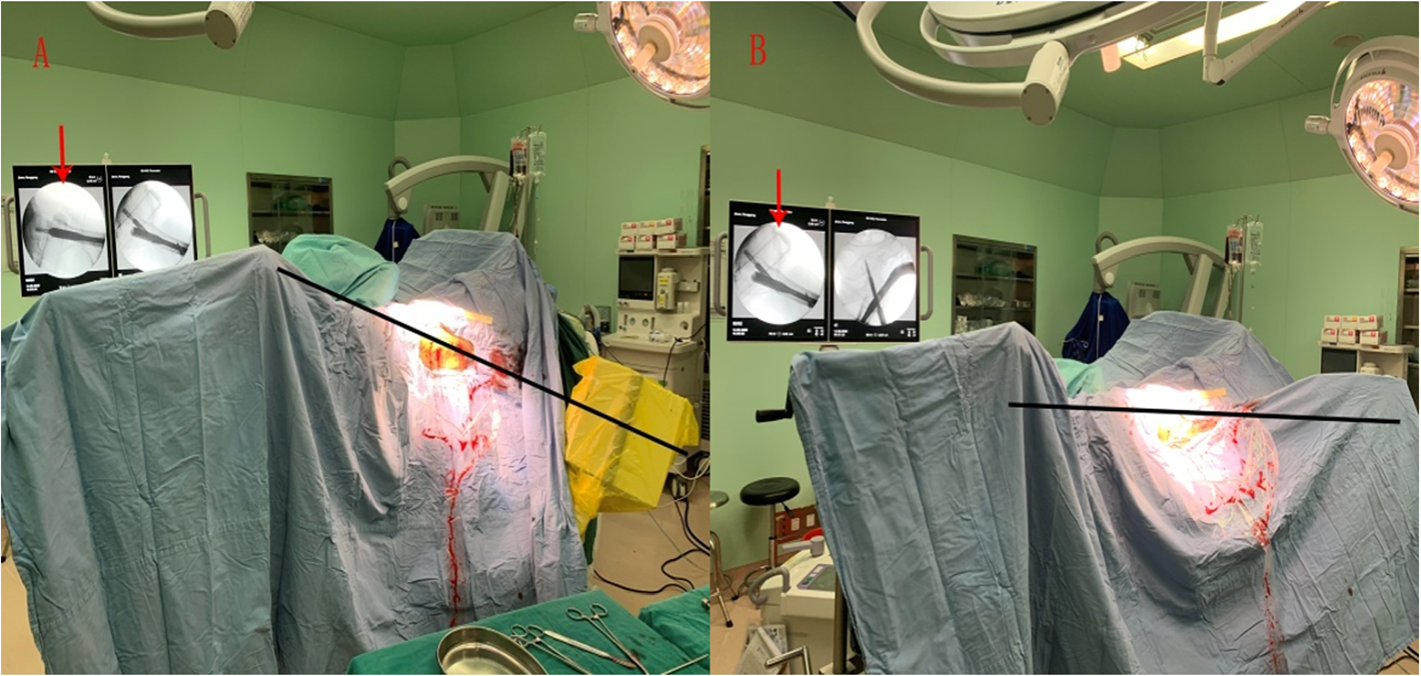
Intraoperative fluoroscopy of the 30 ° oblique view. **a**: Firstly, the standard lateral view (true sagittal) of the femoral neck was got i.e. the helical blade in the femoral head was aligned in a straight line with the nail in the femur medullary canal (set as 0°). **b**: The C-arm was rotated 30° lower to get the new anteromedial oblique tangential view

After completion of the nail fixation, we firstly got the standard lateral view (true sagittal) of the femoral neck i.e. the helical blade in the femoral head was aligned in a straight line with the nail in the femur medullary canal (set as 0°). Then the C-arm was rotated to get the new anteromedial oblique tangential view, usually at 30 °C-arm rotations (Fig. 2).

Post-operative CT-scanning was performed within one week and 3D images were reformed. The 3D-CT reconstruction images were considered the gold standard as they can be rotated 360°to observe the complete view of the cortex apposition between the head-neck and shaft fragments. The fracture reduction quality of the anteromedial cortex in 3D-CT was categorized into two types: true anteromedial cortical support (positive and anatomic) or loss of anteromedial cortical support (negative).

Two orthopedic surgeons (1 resident and1 attending) reviewed the intraoperative fluoroscopic and postoperative 3D-CT reconstruction images to evaluate the relationship of the anteromedial cortical apposition between the head-neck and the femoral shaft. If there was any disagreement, the final agreement was achieved via discussion.

The results of intra-operative fluoroscopic and post-operative 3D-CT reconstruction images were compared and statistically analyzed. Chi-square test was done to determine the difference. Statistical significance was defined as *p* < 0.05.

## Results

### Experimental study

The contour of the anteromedial cortex could not be clearly displayed due to the overlapping of the greater trochanter, the intertrochanteric line and the anterolateral tubercle on the standard femoral neck lateral view. After internally rotating the C-arm to 30°, the overlapping shadows (mostly by the anterior part of the greater trochanter and the anterolateral tubercle of the intertrochanteric line) were completely eliminated, and the tangential projection of the anteromedial cortex was clearly displayed.

**Fig. 3:**
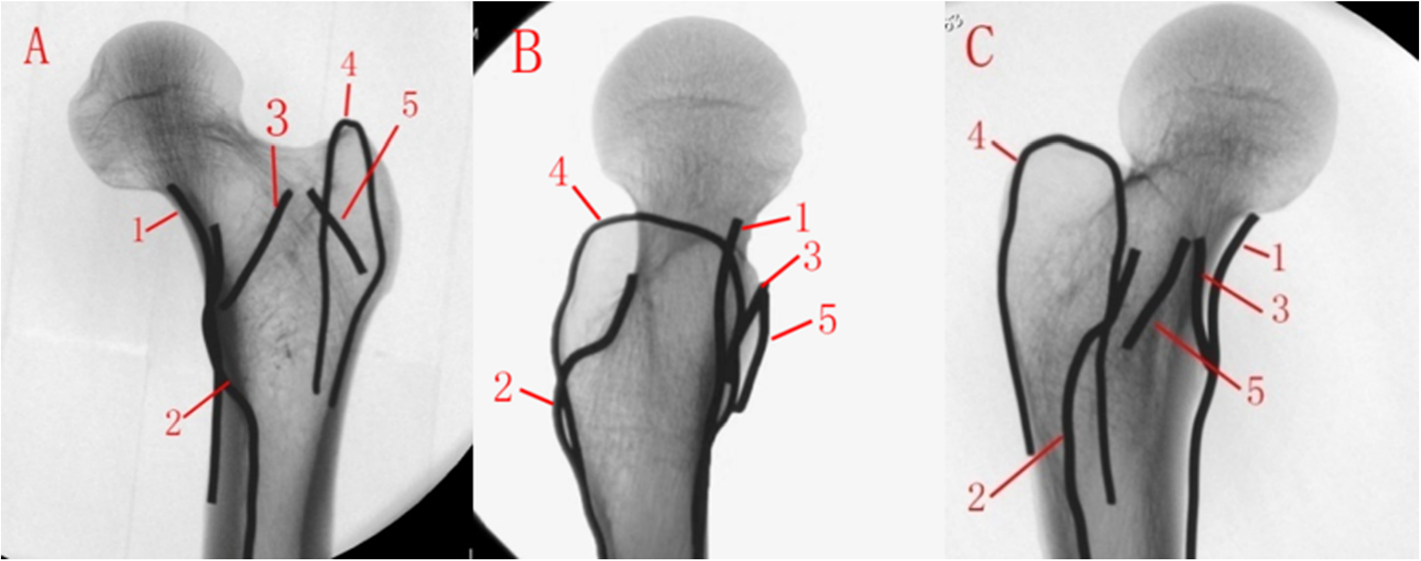
Fluoroscopy of proximal femur. **a**. APview; **b**. lateral view; **c**.30° anteromedial oblique view. Anatomical landmarks: 1. Anteromedial cortical tangent line, 2. Lesser trochanter, 3. Intertrochanteric line, 4. Greater trochanter, 5. Anterolateral tubercle

The fluoroscopic images of the proximal femur with 5 steel marks in AP, lateral and oblique tangential views are shown in Fig. 3.

### Clinical study

The evaluation of the cortical apposition observed in the intra-operative fluoroscopic images were noted to be 79 positive, 19 neutral and no negative positions in the AP view,two positive, 68 neutral and 28 negative positions in the lateral view, and 22 positive, 51neutral and25 negative positions in the oblique view respectively.

The 3D-CT reconstruction images revealed that final anteromedial cortical contact (positive and anatomic) was obtained in 62 cases (63.3%), while lost (true negative) in the remaining 36 cases (36.7%).For the true anteromedial cortical support group, 46cases (46/62, 74.2%) showed a little bit of anterior shift of the inferior cortical spike by flexion rotation of the head-neck fragment (less than 15 degrees), which was an important mechanism of anteromedial cortex-to-cortex buttress.

The overall coincidence rate between intra-operative fluoroscopic and post-operative 3D-CT imageswas63.3% (62/98) in the AP view,79.6% (78/98) in the lateral view, and 86.7% (85/98) in the oblique view (Table 1) respectively. The overall coincidence rate in different fluoroscopic views showed the significant difference (*p* < 0.001). For coincidence between the intra-operative oblique fluoroscopic and post-operative 3D-CT images, 21 cases (21/22, 95.5%) were consistent in the positive group, 40 cases (40/51, 78.4%)in neutral group and 24 cases (24/25, 96%) in negative group. The results demonstrated that a neutral oblique cortex apposition in fluoroscopy would have a post-operative change rate (20%) to negative category.

**Table 1:** Difference inanteromedial cortical contact between intra-op fluoroscopy and post-op 3D CT

Intra-op fluoroscopy				Post-op 3D CT	
AP view	Lateral view	Oblique view	No. of cases	True cortical contact no. (%)	Loss of cortical contact no. (%)
positive	positive	positive	2	2 (100%)	0 (0%)
positive	neutral	positive	16	16 (100%)	0 (0%)
positive	neutral	neutral	35	31 (88.6%)	4 (11.4%)
positive	neutral	negative	5	1 (20%)	4 (80%)
positive	negative	positive	4	3 (75%)	1 (25%)
positive	negative	neutral	8	3 (37.5%)	5 (62.5%)
positive	negative	negative	9	0 (0%)	9 (100%)
neutral	neutral	neutral	8	6 (75%)	2 (25%)
neutral	neutral	negative	4	0 (0%)	4 (100%)
neutral	negative	negative	7	0 (0%)	7 (100%)
			Total:98	62	36

As both positive and smooth neutral cortical appositions in fluoroscopy are accepted in the operation, we also made a comparison between negative group and non-negative group (Table 2). It is demonstrated that the oblique cortical position determined whether the final anteromedial cortical contact is present or not. If a negative oblique position was seen on intra-operative fluoroscopy, regardless of the AP and lateral views, it was highly predictive of a final loss of cortical support as demonstrated by 3D CT (24/25 cases, 96%). In contrast, non-negative oblique cortical position was highly associated with a true cortical contact, as demonstrated by 3D CT (61/73 cases, 83.6%). This was an independent predictive factor as significant difference was showed in oblique view(p<0.001).

**Table 2:** Four combinations patterns of anteromedial cortical contact between intra-op fluoroscopy and post-op 3D CT

Intra-op fluoroscopy				Post-op 3D CT	
AP view	Lateral view	Oblique view	No. of cases	True cortical contact no. (%)	Loss of cortical contact no. (%)
Non-negative	Non-negative	Non-negative	61	55 (90.2%)	6 (9.8%)
Non-negative	Non-negative	negative	9	1 (11.1%)	8 (88.9%)
Non-negative	negative	Non-negative	12	6 (50%)	6 (50%)
Non-negative	negative	negative	16	0 (0%)	16 (100%)
			Total:98	62	36

**Fig. 4:**
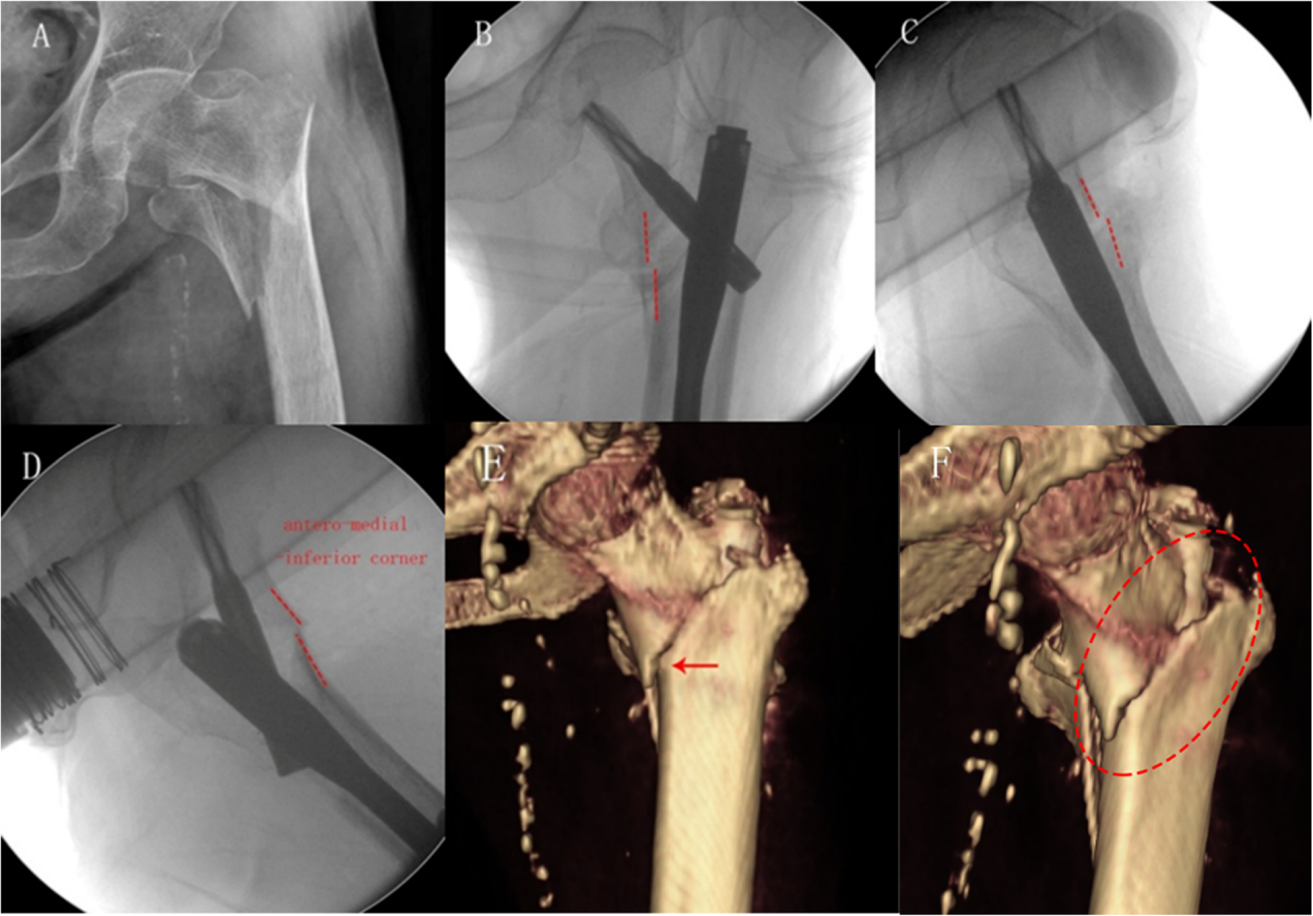
An 84 years old female with pertrochanteric femur fracture (AO/OTA-A2.3). **a**: Preoperative AP view; **b**: Intraoperative AP view; **c**: Lateral view; **d**: 30° anteromedial oblique view showed positive cortical support reduction pattern; **e**:Post-operative 3D-CT image revealed the anteromedial cortex achieved a true contact (positive support). **f**:A slightly flexed rotation of the head-neck fragment resulted in a minor forward shift of the inferior spike, and confirmed stable cortex-to-cortex contact of the anteromedial inferior corner

An illustrative case of the anteromedial oblique fluoroscopy method was shown in Fig. 4.

## Discussion

For the stable reconstructions after fracture reduction and fixation, 5 influencing factors have been summarized, i.e. bone quality (osteoporosis), fragment geometry (comminution), fracture reduction quality, implant selection, and implant placement (TAD/Cal-TAD) [6]. Fracture reduction quality is the first pre-requisite for the treatment of the pertrochanteric fracture [7]. Insufficient fracture reduction can result into the incorrect position of the lag screw/helical blade and even the failure of the mechanical stability of the internal fixation system which may impair the patients’ functional outcomes [8].

Previously, a lot of fracture reduction quality criteria have been described in the literature. These criteria include two aspects which are Garden alignment and fragment displacement. The alignment is similar, and the Garden index is used. However, the displacement criteria are different among different authors. For example, greater than 5 mm by Sernbo [9](1988), 4 mm by Baumgaetner [10] (1995), and 20% by Fogagnolo [11] (2004). Fracture reduction quality is categorized as good (presence of both alignment and displacement criteria), acceptable (either one criterion) or poor (absence of both criteria). In practice, as the detached lesser trochanteric fragment in unstable fracture patterns is usually not reduced and secured clinically by most implants [12], it is hard to achieve “good” reduction quality. Most patients can only meet an “acceptable” quality according to Baumgaertner’s criteria.

In 2015, Chang et al. [5] put forward the new reduction quality criteria: (1) alignment (Garden index): normal or slight abduction of the neck-shaft angle in the AP view, and less than 20 °in the lateral view; (2) apposition: the medial cortices positive or neutral to each other in the AP view; and the anterior cortices smooth or neutral in contact to each other in the lateral view. The reduction quality could also be divided into three grades: good, acceptable and poor. In 2019, Mao et al. [13] explored the reliability of predicting mechanical complications by using Baumgaertner’s criteria and Chang’s criteria. The authors believed that the Chang’s criteria which emphasized anteromedial cortical contact was more reliable and effective than the Baumgaertner’s criteria in predicting postoperative mechanical complications and worthy of clinical applications. Biomechanical [14] and clinical [15] researches have confirmed that the anteromedial cortical contact can not only share stress load from the internal fixation device (mechanical role), but also can promote close contact and fracture healing at the fracture sites (biological role), both of which are benefit for maintaining the normal femoral neck length and the neck-shaft angle.

The anteromedial cortical contact is the key element in evaluation of fracture reduction quality. In 2018, Chang et al. [16] conducted a study on the medial and anterior cortical reduction quality by intraoperative AP and lateral fluoroscopic images and compared it with the post-operative 3D-CT reconstruction images to judge the accuracy of intraoperative fluoroscopic alignment of anteromedial cortex indirectly. The results showed that a positive AP position combined with a positive/neutral lateral position had a high predictive value of the cortical support as confirmed by 3D CT reconstruction images (15/17, 88.2%). While the negative lateral position observed on intra-operative fluoroscopy, regardless of the AP position, was predictive of the final loss of cortical support as demonstrated by 3D-CT reconstruction images (6/7, 85.7%).

Intraoperative flouroscopy allows the surgeon to do a live rotation arc across the fracture to verify reduction. It is ideal to see the AP, lateral and all the obliques to verify fracture reduction is maintained. However, full range view of 360 degrees is impossible in daily practice, and has a greater amount of radiation exposure.

It is apparent that the detection of an anteromedial tangential view without obstruction of other structures may probably enhance the accuracy in interpretation of the relationship between cortices of the head-neck and the shaft fragments. In this study, five anatomical landmarks on the proximal femur were marked with steel wires: the Greater trochanter, the Lesser trochanter, the Intertrochanteric line, the Anterolateral tubercle and the Anteromedial cortical tangent line. After obtaining the standard lateral view of the femoral neck by intra-operative fluoroscopy, we gradually rotated the machine internally to eliminate the overlapping structures on the anterior aspect. We found out that with 30°of rotation, a clear tangential projection of the anteromedial cortex can be obtained.

Clinically, the anteromedial oblique fluoroscopic view of 30° was used to evaluate the alignment of the antero-medial-inferior corner cortex. Compared with AP and lateral views, the oblique fluoroscopic view had a higher accuracy in evaluation of anteromedial apposition. The final loss of cortical contact in 3D-CT was seen in almost all in the negative groups (24/25,96%). This suggested that the negative reduction of the anteromedial cortices needs further re-correction during the operation. Postoperative 3D-CT images revealed that approximately 20%(10/51) of cases in neutral group (observed via oblique fluoroscopic view) did not attain the anteromedial cortical support in the final. In reality, the neutral group shown on intra-operative fluoroscopy may actually have three sub-patterns: 1) an exact anatomic position, 2) a slightly positive position and 3) a slightly negative position. However, as the intra-operative fluoroscopic image resolution is limited, 2-mm cortical steps may not be able to be distinguished clearly. Thus, it becomes unfavorable for a slightly negatively positioned pattern. As the head-neck fragment gets influenced by postoperative muscle contraction and weight-bearing, a slight negative position might become a true negative in postoperative period and finally leading to the loss of the cortical contact.

Several factors may affect the rotation of the head-neck fragment: (1) it is difficult to accurately reduce the anterior and medial cortices in closed reduction, which means exact anatomic reduction of the anteromedial cortices is rare. (2) the intra-operative fluoroscopic image resolution is limited with two dimensional image, which is difficult to observe the rotation of the head-neck fragment. (3) high speed driving of guide pin or reamer, wrenching in the lag screw, or hammering in the helical blade may cause rotation of the head-neck fragment. (4) post-operative axial telescoping may be interfered by head-neck rotation and tilting [17]. Our experience showed that a slightly flexed rotation of the head-neck fragment (less than 15 degrees) is preferable for anterolateral cortical support (46/62, 74.2%).

There are some limitations in this study: (1) this is a retrospective case series study. (2) The anteromedial cortical alignment was evaluated after the cephalomedullary nail insertion. At this point, further corrections of the fracture reduction were impossible. (3) this is only a radiographic comparison study and no clinical outcomes during follow-up periods were described.

## Conclusions

Intra-operative anteromedial oblique view of 30° can eliminate the occlusive overlapping shadows. It is a very useful technique in the evaluation of the anteromedial cortical reduction quality,and has a high prognostic value with the final postoperative 3D-CT.

## Data Availability

The datasets used and analysed during the current study available from the corresponding author on reasonable request. If requested (please contact shiminchang11@aliyun.com).

## Abbreviations

AP: Antero-posterior
AO/OTA: AO Foundation and Orthopaedic Trauma Association
3D-CT: Three dimention-computed tomography
TAD/cal-TAD: Tip apex distance/calcar referenced tip-apex distance
